# Supplementary material for: Infection Manager System (IMS) as a new hemocytometry-based bacteremia detection tool: A diagnostic accuracy study in a malaria-endemic area of Burkina Faso
Source: PLoS Negl Trop Dis. 2021 Mar 1;15(3):e0009187. doi: 10.1371/journal.pntd.0009187 (PMC7951874; doi:10.1371/journal.pntd.0009187)
Supplement: S1 Text — (DOCX) [file pntd.0009187.s001.docx]

S1 Data. Sample collection, case definitions and diagnostic procedures

Sample collection

In all enrolled children, blood cultures were taken together with an ethylenediaminetetraacetic acid (EDTA)-anticoagulated venous blood sample for a complete blood count, malaria slide and malaria rapid diagnostic tests (RDT). Left-over plasma and whole blood samples were stored at -80 ^o^C for retrospective PCR malaria and PCR on selected bacterial pathogens (see below). A nasopharyngeal swab was taken in all children and stored at -80^o^C. A multiplex PCR was retrospectively performed on the swabs from children with respiratory tract symptoms and negative results for the malaria blood smear, blood cultures and bacterial PCR. Blood cultures were repeated during hospitalization upon indication. Additional samples such as urine were taken at the discretion of the treating physician if clinically indicated.

Urine samples were taken in case of clinical dysuria, macroscopic hematuria or polyuria. Samples were tested for recent antibiotics use. After a dipstick analysis 10 µL urine was incubated on McConkey agar and CLED. Stool samples were taken in case of persistent watery diarrhea. Samples were diluted in a 1:5 ratio in normal saline and examined by direct microscopy for intestinal parasites and yeast. Diluted samples were then incubated on MacConkey agar and selenite broth. A subculture of selenite broth was then incubated on Salmonella-Shigella agar and McConkey. Pus was sampled using a syringe (abscess) or sterile swab (superficial infection) and incubated on blood agar. Cerebrospinal fluid (CSF) was sampled in case of clinical suspicion of meningitis. The blood cell count in CSF was measured by hematology analyzer (Sysmex XN-1000), after which a rapid diagnostic test (Pastorex Meningitis) was done (Clinical Diagnostics, Bio-Rad, California, USA) and consequently incubated on blood agar. All cultured isolates were identified to the species level using the API system (BioMerieux).

In case of suspicion of a newly diagnosed HIV or tuberculosis, patients were referred to the national diagnostic centers for HIV or tuberculosis located in the compound of CMA. Additional tests including blood chemistry or imaging diagnostics such as chest X-ray or abdominal echography were done on indication. Nasopharyngeal swabs, isolated bacteria and stored aliquots were shipped to Radboudumc Nijmegen, the Netherlands, on dry ice for malaria and bacterial PCR analysis.

Laboratory procedures

**Malaria diagnostics** included malaria microscopy and malaria PCR. Thick and thin blood films were stained with 3% Giemsa solution and examined for presence of *Plasmodium* parasites as previously reported [1]. Results were expressed as asexual parasites per microliter (p/µl) using the patient’s white blood cell (WBC) count. Slides were examined by two independents qualified microscopists. Presence of one or more *Plasmodium* parasites was considered as a positive test. In case of discrepancies between the two readers results (*e.g.* discrepancy between positive and negative slides, a more than 1 log difference in parasite density, and discrepancy in *Plasmodium* species) a third microscopist’s reading blinded to the results of previous readers was required. The final result was the average of the two closest reader’s results.

**Blood culture** was performed for each participant using 1-3 ml blood for children (BD BACTEC Peds Plus™, Becton Dickinson (BD), Sparks, Maryland, USA) and 10 ml blood for adults (BD BACTEC Aerobic medium™, BD). Culture bottles were incubated in a BACTEC 9050 (BD) for 5 days. If flagged positive the sample was sub-cultured appropriate agar. General media such as Columbia agar+5% sheep blood, or chocolate supplemented agar (bioMérieux, Marcy-l'Etoile, France) were used and incubated at 35 – 37°C for 24 hours under CO2 condition. In addition, selective media such as Eosin-Methylene Blue (EMB) agar and mannitol salt agar (bioMérieux, Marcy-l'Etoile, France) were plated and incubated aerobically at 35 – 37°C for 24 hours, depending on Gram staining results. Isolates were identified by standard microbiology and biochemical methods (API strips, bioMérieux Marcy-L’Etoile, France). Antibiotic susceptibility testing was performed by disk diffusion method and by determination of minimal inhibitory concentration (MIC) values using E-test macromethod (bioMérieux) according to CLSI criteria.

**Bacterial PCR** was done on plasma and whole blood for *Streptococcus pneumoniae*, *Haemophilus influenzae* and *Staphylococcus aureus,* and *Salmonella* spp. Respectively. Stored plasma samples were thawed on ice and 400 µl from each sample was used for DNA isolation using the MagNA Pure LC 2.0 (Roche). For the identification of *S. pneumoniae* a Taqman assay was performed (PMID 17537936) and for the identification of *S. aureus* and *H. influenzae* a SYBR Green assay was performed (PMID 20226325 en 21276750). Whole blood sample was used for the identification of *Salmonella* spp. in the laboratory of the Department Medical Microbiology at Radboudumc, according to the local laboratory standards procedures*.* In short; from each sample 200 µL was used for DNA isolation using the MagNA Pure 96 system. Real time PCR was used with Taqman probes to target Salmonella as described elsewhere [2]. In 184 (22.1%) samples, the available volume was less than 200 µL (range 50-200 µL); these samples were not excluded from analyses.

**Quantitative malaria PCR (qPCR)** targeting the multicopy 18S rRNA gene was performed at the Department Medical Microbiology at Radboud university medical center using 200 µl of whole blood as previously described [3].

**Nasopharyngeal swabs** were analysed for patients of <15 years old with a clinical suspicion of a respiratory tract infection in the absence of a positive malaria microscopy and blood culture. The presence of pathogens was assessed using the ePlex system multiplex PCR on 23 different viral (Adenovirus, Coronavirus [229E, HKU1, NL63, OC43, MERS coronavirus], Human bocavirus, Human metapneumovirus, Human rhinovirus/enterovirus, Influenza virus A [H1,2009 H1N1,H3], Influenza virus B, Parainfluenza virus [1,2,3,4], Respiratory syncythial virus [A,B]) and 4 different bacterial (*Bordetella pertussis, Chlamydophila pneumoniae, Legionella pneumophila, Mycoplasma pneumoniae*) (GenMark Diagnostics, Inc, Carlsbad, CA) as described in detail elsewhere [4]. Isolation of bacterial isolates were not considered for the diagnostic classification scheme since the distinction between pathogens and colonizers is very difficult to make.

**PCT and CRP** batch tested according to the manufacturer’s instructions using enzyme-linked immunosorbent assays (ELISA) (Procalcitonin, (Invitrogen, Thermo-Fisher, Waltham, Massachusetts, United States) and CRP 96 DET (Tecan, Männedorf, Switzerland) respectively).

Case definitions

Malaria was defined as the presence of one or more parasites in malaria microscopy or a malaria qPCR result of over 0.5 p/µL or a malaria qPCR of more than 0.05 p/µL in a patient that recently used anti-malarials. Clinical malaria was defined as malaria in the presence of a clinical presentation fitting with malaria. Bacteraemia was defined as a blood culture yielding a pathogenic microorganism or a positive blood bacterial PCR in combination with clinical suspicion of bacteraemia. Coagulase negative staphylococci (CNS) and gram-positive bacilli were considered contaminants unless stated otherwise. Urinary tract infection was defined as a culture yielding pure growth of 10^5^ colonies or more, (ii) a pure culture of <10^5^ colonies in the presence of recent antibiotics use, (iii) a urine dipstick positive for nitrite and leukocytes in the presence of recent antibiotics use. Gastroenteritis was defined as a stool culture yielding *Salmonella spp.*, *Shigella spp.* or enterotoxic *Escherichia coli*. Bacterial meningitis was defined as a positive rapid diagnostic test or CSF culture yielding any microorganism potentially involved in meningitis. Pus and clinical abscesses were considered a sign of localized invasive bacterial infection. Newly diagnosed HIV was defined as a positive rapid diagnostic test in a patient who had previously not been tested, or had previously tested negative for HIV. Tuberculosis (TB) was defined as a positive Ziehl-Neelson stain on at least one of three sputum cultures, or a clinical suspicion with radiological confirmation (e.g. miliary TB). Respiratory tract infections were defined based on the clinical picture combined with nasopharyngeal swab result and chest X-ray, the latter two also defining whether and a bacterial or viral origin was present. Viral hepatitis was defined as a positive HBSAg and anti-HBc test. Co-infections were defined as the presence of two or more proven infections in the same patient.

Data collection, quality control and quality assurance

Data were collected on standardized case report forms (CRFs) and entered into a secure database (RedCap, Vanderbilt University, Nashville, USA) after conformity check by a medical doctor. Entered data were checked against the CRFs by a data manager. Approximately ten percent of patient study files were checked by an independent monitor. Results from PCRs and ELISAs were entered into an excel database and merged with the principal database upon completion of inclusion. Laboratory analyses were performed and interpreted by experienced laboratory technicians who were blinded to clinical data. Raw data from the XN-450 was electronically transferred to SYSMEX company once every two weeks for storage. SYSMEX had no access to the clinical data, researchers were blinded to the XN-450 results until all clinical data were locked into a STATA database. Quality control and quality assurance were done in accordance with Good Clinical and Laboratory Practice (GCLP) guidelines.

References

1. Guiraud I, Post A, Diallo SN, Lompo P, Maltha J, Thriemer K, et al. Population-based incidence, seasonality and serotype distribution of invasive salmonellosis among children in Nanoro, rural Burkina Faso. PLOS ONE. 2017;12(7):e0178577. doi: 10.1371/journal.pone.0178577.

2. Malorny B, Paccassoni E, Fach P, Bunge C, Martin A, Helmuth R. Diagnostic real-time PCR for detection of Salmonella in food. Applied and environmental microbiology. 2004;70(12):7046-52. doi: 10.1128/AEM.70.12.7046-7052.2004. PubMed PMID: 15574899.

3. Reuling IJ, van de Schans LA, Coffeng LE, Lanke K, Meerstein-Kessel L, Graumans W, et al. A randomized feasibility trial comparing four antimalarial drug regimens to induce Plasmodium falciparum gametocytemia in the controlled human malaria infection model. eLife. 2018;7. Epub 2018/02/28. doi: 10.7554/eLife.31549. PubMed PMID: 29482720; PubMed Central PMCID: PMCPMC5828662.

4. Nijhuis RHT, Guerendiain D, Claas ECJ, Templeton KE. Comparison of ePlex Respiratory Pathogen Panel with Laboratory-Developed Real-Time PCR Assays for Detection of Respiratory Pathogens. Journal of clinical microbiology. 2017;55(6):1938-45. Epub 2017/04/14. doi: 10.1128/jcm.00221-17. PubMed PMID: 28404682; PubMed Central PMCID: PMCPMC5442551.
