# Supplementary material for: Infection Manager System (IMS) as a new hemocytometry-based bacteremia detection tool: A diagnostic accuracy study in a malaria-endemic area of Burkina Faso
Source: PLoS Negl Trop Dis. 2021 Mar 1;15(3):e0009187. doi: 10.1371/journal.pntd.0009187 (PMC7951874; doi:10.1371/journal.pntd.0009187)
Supplement: S2 Text — (DOCX) [file pntd.0009187.s002.docx]

S2 Data. Technical supplement on the development of the IMS

The XN-series hematology analyzers apply flow cytometry and fluorescence to measure individual cells in peripheral blood. After exposure to a mix of surfactants and staining solution the cells measured individually by a red laser and categorized based on their morphologic structure. Each cell is then plotted on a 3D graph whereby the X-axis represents the cellular granularity and internal complexity of the cell (side scatter light), the y-axis represents the nucleic acid/protein contents (fluorescent light intensity) and a z-axis represents cell volume and shape (forward scatter light).The data is analyzed three dimensionally [1].

The Infection Management System (IMS) is a diagnostic algorithm developed to characterise the cellular reaction to different aetiologies of acute infection. It is anchored on the biological premise that extracellular/intracellular bacteria, viruses and malaria all trigger varying immune responses, which can be characterised by assessing patterns of leukocyte subsets in the peripheral blood. Neutrophil leucocytosis is commonly associated with bacterial infection and lymphocytosis with viral infection; though frequently observed these associations are not absolute. The IMS utilises both conventional and newly developed haematological parameters to evaluate the different patterns of immune activation and estimate the likelihood of the following clinical scenarios: malaria infection, viral infection, bacterial infection, inflammatory response without infection, no immune activation.

Central to the IMS are the Sysmex Haematology Parameters derived from the ‘CBC + WBC differential’. These parameters can be categorized into four groups: neutrophil related parameters, lymphocyte related parameters and monocyte related parameters, which are further detailed below. Other blood cell counts such as platelet count and haemoglobin related parameters are also used in the algorithm.

Neutrophil related parameters

In the leukocyte differential of a healthy adult, neutrophils account for approximately 60% to 70% of leukocytes. They are counted among polymorph nuclear cells (PMN) – also called granulocytes – due to the segmented shape of the nucleus among mature cells. Immature neutrophils are still in the process of developing a segmented nucleus and are therefore often referred to as ‘bands’ or ‘banded neutrophils’.

Neutrophil activation forms the backbone of the early innate immune response. They are typically activated by antigen presenting cells while still in the bloodstream and subsequently migrate to the site of infection within minutes after infection has been established. Their primary objective is to neutralise the infection by phagocytosis, but they also have an immunomodulatory function. Activated neutrophils secrete a variety of pro-inflammatory cytokines which helps dendritic cells, monocytes and lymphocytes to decide on how to proceed and maintain the immune response [2-4].

Acute infection is characterised by the release of neutrophils from the bone marrow into the peripheral bloodstream, creating an increase in the absolute number and percentage of neutrophils, and causing a ‘left-shift’; an increase in immature cells such as banded neutrophils. A previously proposed marker founded on the premise that neutrophils increase in early infection is the neutrophil-to-lymphocyte Ratio (NLR), whereby an NLR of 10 or more has been associated with bacterial infections [5-7]. The neutrophil-to-lymphocyte ratio is one of the variables incorporated into the IMS.

Absolute and relative neutrophil counts are standard issue in a leukocyte differential analysis. The novel neutrophil related parameters include the absolute immature granulocyte (IG) count and IGs as a percentage of total leukocyte count, the neutrophil reactivity index (NEUT-RI) and the neutrophil granularity index (NEUT-GI). As previously described, the novel series haematology analysers differentiate cells according to their fluorescence signal intensity, their size and the complexity of their internal structure [8].

Immature Granulocytes (IG) include metamyelocytes, myelocytes and promyelocytes which are precursor cells to granulocytes. Banded neutrophils are not included in the group of Immature Granulocytes. IG’s are different from their mature counterparts in that they are typically larger and have a larger nucleus. Previous studies have demonstrated that haematology analysers are capable of reliably differentiating them within a leukocyte differential, in reference to microscopy [9].

Activation of neutrophils leads to morphological changes in the cell structure: cytokine production increases their cytoplasmic activity, toxic granulation and vacuolisation increase their granularity and changes in the cell-wall lipid rafts and upregulation of membrane receptors lead to an increased deformability, increased motility and increased adhesion capacity; all of which are needed for an effective response to the encountered pathogen.

These morphological changes increase the intensity of the fluorescence signal, which allows for differentiation of activated cells from inactivated cells. The parameters NEUT-RI and NEUT-GI reflect these changes; NEUT-RI represents increases in cytoplasmic activity and NEUT-GI represents an increase in intracellular cytokine containing vacuoles. Previous reports demonstrate the validity of NEUT-RI and NEUT-GI as markers of neutrophil activation [8] and demonstrated their potential for detection of bacterial infection in patients with sepsis[10]. Since neutrophil activation is an indicator of an early innate immune response, both NEUT-GI and NEUT-RI are increased in acute infection while presence of IG’s in the peripheral blood stream are a sign of either acute infection/inflammation or haematological malignant disease.

Monocyte related parameters

Monocytes account for approximately 4-8% of cells in a healthy adults’ leukocyte differential. Together with PMNs they make up the cellular component of the innate immune response. Once activated, monocytes are recruited from the peripheral blood to the site of inflammation, where they are differentiated into macrophages (phagocytic cells) or dendritic cells (antigen presenting cells). They have three primary target functions; first, to phagocytose the present pathogens, second, to modulate the immune response by producing pro- or anti-inflammatory cytokines and third, to antigen presentation to lymphocytes and other monocytes in lymph nodes to activate the adaptive immune response. Where neutrophils can be considered the brute workforce of the innate immune system, monocytes are typically involved in regulation and control of the larger immune response. Monocytes are not a homogenous population of cells but rather show different phenotypes which feature different functions and abilities [11, 12]. These cells are typically referred to as classical and non-classical monocytes.

Just like activated neutrophils, activated monocytes are also characterized by an increased deformability, motility and adhesion capacity, and an increase in granularity and vacuolisation. As described, under normal conditions monocytes circulate the peripheral blood for several days before migrating into tissue to differentiate [13]. During inflammation and infection this process is considerably accelerated, which leads to a decline of the absolute monocyte count in the peripheral circulation. The high splenic reserves as well as the bone marrow capacity to rapidly proliferate monocytes quickly replenishes their numbers to normal or increased values as a result of pro-inflammatory cytokines and other molecules originating from the infecting microorganism.

The novel parameter Reactive Monocytes (RE-MONO) provides the absolute number and percentage of activated monocytes. Whilst resembling higher activation levels [12] and proven to be patrolling and featuring antiviral roles, non-classical monocytes are not typically referred to as activated monocytes as a result of their capacity to mature into multiple cell lines. However, since they resemble the biological meaning of RE-MONO and are increased in infection and inflammation, they are included in the RE-MONO parameter. The validity of RE-MONO as a marker for activated monocytes has been previously demonstrated in ex-vivo experiments [8]. Activated monocytes are typically found in acute severe infections.

Lymphocyte related parameters

Lymphocytes are a subset of leukocytes with a very large nucleus and little cytoplasm, which are mainly found in the lymph fluid and lymph nodes. They encompass different cell lines, most importantly 1) Natural Killer (NK) cells, which play an important part in the innate response against viruses and 2) T-cells and B-cells which form the core of the cellular (T-cells) and humoral (C-cells) adaptive immune response. Both T-cells and B-cells can be further subdivided into cell-subsets with different functionality. Lymphocytes account for approximately 30% of cells in the peripheral leukocyte differential of a healthy adult. Due to their common characteristics it is difficult to differentiate lymphocyte cell-lines based on morphology. In clinical care, immunophenotyping can be used to assess the presence of cell-wall receptors which are specific to the various cell-lines within the lymphocyte group.

The new parameter RE-LYMPH represents reactive lymphocytes; these encompass activated B-cells, T-cells and natural killer cells which have a fluorescent signal which is higher than the regular lymphocyte population. AS-LYMPH refers to antibody secreting B-lymphocytes – also called plasma cells – and is a marker of the humoral immune response. Antibody secreting lymphocytes have a higher fluorescent signal and are more complex than any of the other leukocyte subsets and therefore show as a separate population of cells in the scatterplot. The presence of RE-LYMPH is a sign of viral infection whereas the presence of AS-LYMPH is a sign of advanced infection with a previously encountered pathogen.

Outcome parameters of the IMS: flags and likelihood scores

First, the IMS provides a complete blood count (CBC) with regular leukocyte differentiation including absolute numbers and percentages of neutrophils, lymphocytes, monocytes, basophils and eosinophils, which is one of the most frequently requested diagnostic tests in patients with acute febrile illness.

Second, the IMS provides a calculated likelihood score for bacterial infection and viral infection. The likelihood score for each flag is comprised of a combination of variables: abnormal (either increased or decreased) cell counts or cell ratios account for points in the likelihood scores whereby different parameters are weighted differently for the various flags.

Likelihood scores range from 0-300 for viral and bacterial infections. A score of 0 represents a complete mismatch while a score of 300 represents a complete match with the reference pattern (e.g. bacterial or viral infections). Scores of less than 20 in all categories show an absence of any immune response. If none of the scores are 50 or higher, the IMS classifies inflammation of unknown origin. Equal likelihood scores between bacterial and viral flags indicate a possible co-infection. A likelihood score of 100 or more is considered a clear positive. In case inflammation is flagged but none of the likelihood scores reach 100 or more, the IMS selects the highest score of as most likely cause of infection; except when scores are close together in which case both flags will appear.

For the analysis of the current dataset, inflammation matching bacterial infection was defined as a bacterial likelihood score of 100 or more, or a bacterial likelihood score of 50 or more where both the viral and malaria likelihood scores are lower than the bacterial likelihood score. If the bacterial likelihood score falls between 50 and 100, and the viral score exceeds the bacterial score, the IMS will indicate a viral infection. If the bacterial likelihood score is higher than 100, the IMS will indicate a bacterial infection even if the viral score exceeds 100 as well. In these cases, both the bacterial and the viral flag will appear. The prototype used in this study also had a functionality to detect malaria infections based on the immune response profile, but that functionality is not yet fully developed.

Development of the algorithm

The conception of the IMS was the result of analysis of data principally collected to improve the detection of malignant haematological diseases by haematology analysers. Datasets containing patients with and without malignant haematological disease were analysed to differentiate malignant from reactive lymphocytosis. During this process the novel lymphocyte parameters were developed in order to facilitate this process. Reactive lymphocytosis in this population was predominantly the result of viral infections. The populations used were predominantly Caucasian and Asian in origin, these populations formed the basis of the viral flag for the IMS, later supplemented with data from Indonesia where arboviruses are common.

In order to develop the intensive care infection score (ICIS), databases of hospitalized patients with bacteraemia were collected from several hospitals in Europe and the United States. Monocyte and neutrophil activation parameters were subsequently developed and led to the development of the first bacterial IMS flag.

The prototype of the malaria flag resulted from the development of the XN-30 malaria diagnostics tool as previously published. Samples were collected from the Asian and African continents and analysed. While the sexual stages of plasmodium can quite reliably be detected by routine Sysmex haematology analysers, infections with *P. falciparum* remain challenging.

The first prototype of the IMS was therefore created on a multitude of cohorts, principally collected for other purposes. In order to develop the IMS algorithm, all the above cohorts were combined with a cohort of healthy controls. Patterns of infection were assessed and compared with healthy controls and patients with different causes of infection. The resulting algorithm was then tested on the Indonesian cohort and subsequently on the current cohort. The current study was the first large cohort of paediatric patients to which the IMS was exposed.

Future development of the IMS

The IMS has been visualized as a tool to assist healthcare workers in their decision to either prescribe or withhold antibiotics in patients presenting with acute febrile illness in second- and third line healthcare facilities such as hospitals and large healthcare centers, comparable to CRP. Since it is an algorithm, it has the capacity to improve with time as additional data is fed into the algorithm.

As described in the main text, Sysmex is simultaneously developing a second technology, which uses a different wavelength of laser and different reagents, to directly detect parasitized erythrocytes. This technology has proven to be at least as effective as high quality microscopy (gold standard) and more accurate than rapid diagnostic tests based on HRP2 and pLDH [14]. The ultimate goal is to develop a small hematology analyzer which combines both techniques and is therefore able to reliably provide information on the presence of malaria parasites in the peripheral circulation, as well as on the likelihood of a bacterial infection.

References

1. Novel haematological parameters for rapidly monitoring the immune system response [Internet]. 2017. Sysmex white paper Infection/inflammation

2. Bennouna S, Bliss SK, Curiel TJ, Denkers EY. Cross-talk in the innate immune system: neutrophils instruct recruitment and activation of dendritic cells during microbial infection. Journal of immunology (Baltimore, Md : 1950). 2003;171(11):6052-8. Epub 2003/11/25. doi: 10.4049/jimmunol.171.11.6052. PubMed PMID: 14634118.

3. Schmielau J, Finn OJ. Activated granulocytes and granulocyte-derived hydrogen peroxide are the underlying mechanism of suppression of t-cell function in advanced cancer patients. Cancer Res. 2001;61(12):4756-60. Epub 2001/06/19. PubMed PMID: 11406548.

4. Wittamer V, Bondue B, Guillabert A, Vassart G, Parmentier M, Communi D. Neutrophil-mediated maturation of chemerin: a link between innate and adaptive immunity. Journal of immunology (Baltimore, Md : 1950). 2005;175(1):487-93. Epub 2005/06/24. doi: 10.4049/jimmunol.175.1.487. PubMed PMID: 15972683.

5. Loonen AJM, de Jager CPC, Tosserams J, Kusters R, Hilbink M, Wever PC, et al. Biomarkers and Molecular Analysis to Improve Bloodstream Infection Diagnostics in an Emergency Care Unit. PLOS ONE. 2014;9(1):e87315. doi: 10.1371/journal.pone.0087315.

6. Zahorec R. Ratio of neutrophil to lymphocyte counts-rapid and simple parameter of systemic inflammation and stress in critically ill. Bratislavske lekarske listy. 2001;102(1):5-14.

7. Naess A, Nilssen SS, Mo R, Eide GE, Sjursen H. Role of neutrophil to lymphocyte and monocyte to lymphocyte ratios in the diagnosis of bacterial infection in patients with fever. Infection. 2017;45(3):299-307. Epub 2016/12/19. doi: 10.1007/s15010-016-0972-1. PubMed PMID: 27995553.

8. Linssen J, Aderhold S, Nierhaus A, Frings D, Kaltschmidt C, Zanker K. Automation and validation of a rapid method to assess neutrophil and monocyte activation by routine fluorescence flow cytometry in vitro. Cytometry Part B, Clinical cytometry. 2008;74(5):295-309. Epub 2008/04/24. doi: 10.1002/cyto.b.20422. PubMed PMID: 18431775.

9. Briggs C, Kunka S, Fujimoto H, Hamaguchi Y, Davis BH, Machin SJ. Evaluation of immature granulocyte counts by the XE-IG master: upgraded software for the XE-2100 automated hematology analyzer. Lab Hematol. 2003;9(3):117-24. Epub 2003/10/03. PubMed PMID: 14521317.

10. Urrechaga E, Boveda O, Aguirre U. Role of leucocytes cell population data in the early detection of sepsis. Journal of clinical pathology. 2018;71(3):259-66. Epub 2017/08/20. doi: 10.1136/jclinpath-2017-204524. PubMed PMID: 28821583.

11. Ziegler-Heitbrock L. The CD14+ CD16+ blood monocytes: their role in infection and inflammation. J Leukoc Biol. 2007;81(3):584-92. Epub 2006/12/01. doi: 10.1189/jlb.0806510. PubMed PMID: 17135573.

12. Ziegler-Heitbrock L, Ancuta P, Crowe S, Dalod M, Grau V, Hart DN, et al. Nomenclature of monocytes and dendritic cells in blood. Blood. 2010;116(16):e74-80. Epub 2010/07/16. doi: 10.1182/blood-2010-02-258558. PubMed PMID: 20628149.

13. van Furth R, Cohn ZA. The origin and kinetics of mononuclear phagocytes. J Exp Med. 1968;128(3):415-35. Epub 1968/09/01. doi: 10.1084/jem.128.3.415. PubMed PMID: 5666958; PubMed Central PMCID: PMCPMC2138527.

14. Post A, Kabore B, Reuling IJ, Bognini J, van der Heijden W, Diallo S, et al. The XN-30 hematology analyzer for rapid sensitive detection of malaria: a diagnostic accuracy study. BMC medicine. 2019;17(1):103. Epub 2019/05/31. doi: 10.1186/s12916-019-1334-5. PubMed PMID: 31146732; PubMed Central PMCID: PMCPMC6543632.
