# Supplementary material for: Infection Manager System (IMS) as a new hemocytometry-based bacteremia detection tool: A diagnostic accuracy study in a malaria-endemic area of Burkina Faso
Source: PLoS Negl Trop Dis. 2021 Mar 1;15(3):e0009187. doi: 10.1371/journal.pntd.0009187 (PMC7951874; doi:10.1371/journal.pntd.0009187)
Supplement: S1 Table — (DOCX) [file pntd.0009187.s003.docx]

**S1 Table** **. Diagnostic classification schema with criteria for the different infections.**

| **Diagnosis** | **Clinical presentations needed for diagnosis** | **Diagnostics needed for diagnosis** | **Classification** |  |
| --- | --- | --- | --- | --- |
| **Malaria** | | | | |
| Malaria | All presentations | Microscopy with 1 or more parasite | Malaria |  |
|  |  | Malaria PCR with > 50 p/uL and clinical picture of malaria or a malaria qPCR of more than 0.05p/µL in a patient that recently used antimalarials | Malaria |  |
| **invasive bloodstream infection** | | | | |
| Bacteremia | All presentations | Blood culture positive for bacteria other than typical contaminants | Bacterial |  |
|  |  | Blood PCR positive for bacteria in combination with clinical picture | Bacterial |  |
| **Meningitis** | | | | |
| Meningitis | Neck stiffness OR bulging fontanel OR Kernig / Brudzinski OR Convulsions OR Abnormal consciousness | CSF culture or Agglutination (Pastorex) positive for bacteria other than typical contaminants | Bacterial |  |
|  |  | No biological confirmation | Infection of unknown origin |  |
| **Urinary tract infection** | | | | |
| Urinary tract infection | Dysuria, hematuria or polyuria | Pure urine culture with >10^5^ CFU/ml, OR positive nitrite in sample taken after antibiotics use | Bacterial |  |
|  |  | Negative urine culture and dipstick or bacteriuria in presence of confirmed gynecological malignancy. | Infection of unknown origin |  |
| **Infection of skin or soft tissue** | | | | |
| Skin infection (including abcess) | Erysipelas, cellulitis, lymphangitis, osteomyelitis, gangrene or abscesses.  Erythema, pain and swelling of a limb. Clinical diagnosis, no bacteriological confirmation required. | Erysipelas, cellulitis, lymphangitis, osteomyelitis, gangrene or abscesses on inspection | Bacterial |  |
| Osteomyelitis | Pain, swelling of a limb, local tenderness, refusal to move the affected limb | Positive culture of pus or collected liquid, X-ray images of bone lysis | Infection of unknown origin |  |
| Septic arthritis | Painful red swollen joints | Painful red swollen joints | Infection of unknown origin |  |
| **Acute abdominal infections** | | | |  |
| Abdominal abscess | Abdominal tenderness | Echographic confirmation of abdominal abscess | Bacterial |  |
| Acute intra-abdominal infection | Acute abdominal tenderness with passive muscle reflexes | Echographic confirmation of peritonitis, adnexitis, appendicitis | Infection of unknown origin |  |
| **Gastroenteritis** | | | | |
| Parasitic gastroenteritis | Diarrhea > 3 stools/day | Stool culture positive for parasites | Infection of unknown origin |  |
| Bacterial gastroenteritis |  | Stool culture positive for Enterotoxic *E. coli, Salmonella* or *Shigella* species. | Bacterial |  |
| Gastroenteritis of unknown origin |  | No amoeba or bacteria on microscopy and culture. | Infection of unknown origin |  |
| **Viral hepatitis** | | | |  |
| Viral hepatitis | Pain in upper right abdomen | Positive HBsAg and anti-HBc | Viral |  |
| **Upper respiratory tract infection (URTI)** | | | | |
| Rhinitis | Running nose | None | Infection of unknown origin |  |
| Pharyngitis | Throat ache with pharyngeal inflammation | None |  |  |
| Tonsillitis | Throat pain OR pharyngeal redness AND enlarged tonsils | None |  |  |
| Acute otitis media | Ear pain with auricular discharge OR bulging tympanum OR erythematous tympanum | None |  |  |
| **Lower respiratory tract infection (LRTI)** | | | | |
| Bronchitis/ Bronchiolitis | either dry or productive cough; normal breathing rate and normal OR abnormal chest auscultation. | No confirmation | Infection of unknown origin |  |
| Pneumonia | Dry or productive cough with rapid breathing OR chest indrawing/nasal flaring OR dyspnoea OR abnormal chest auscultation | Conscript consolidation, lobular infiltrates or heterogenous opacities on CXR with negative nasopharyngeal swab | Bacterial |  |
|  |  | Clinical signs of lower ARI in absence of 1) viral nasopharyngeal swab AND signs of pneumonia on CXR  2) unavailability of CXR | Infection of unknown origin |  |
|  |  | Nasopharyngeal swab  positive for virus in absence of positive CXR signs | Viral |  |
| **HIV** | | | | |
| HIV | Weight loss OR severe malnutrition OR one or multiple superinfections often correlated to HIV | Positive HIV rapid diagnostic test | Infection of unknown origin |  |
|  |  | Confirmed HIV in combination with other confirmed infection (malaria or viral) | Infection of unknown origin |  |
|  |  | Confirmed HIV in combination with confirmed bacterial infection | Bacterial |  |
| **Tuberculosis** | | | | |
| Pulmonary Tuberculosis | Cough for 14 days or more with weight loss | Positive tuberculosis sputum test OR typical caseating granulomas on CXR | Bacterial |  |
|  |  | Negative tuberculosis sputum test and CXR | Infection of unknown origin |  |
| **Mixed infections** | | | | |
| Malaria + bacterial infection | All presentations | Confirmed malaria + biologically or clinically confirmed bacterial infection | Malaria + bacterial |  |
| **Other cases** | | | | |
| Suspected bacterial infections | Previous antibacterial treatment prior the inclusion (bacterial laboratory test) AND improvement under treatment. | Negative on all microbiological diagnostics. Increase of total white blood and differential cells count in full blood count | Infection of unknown origin |  |
| Suspected viral infections | Fever with painful lymphadenopathy, no improvement under antibiotic treatment | Negative on all microbiological diagnostics with a lymphocytosis and trombocytopenia | Infection of unknown origin |  |
| Malaria + suspected bacterial | Clinically ill with improvement under antibiotic treatment | Confirmed malaria diagnostics with uncharacteristically high leukocyte counts (>80% neutrophils) | Infection of unknown origin |  |
| Infection of unknown origin | Patients with an acute febrile illness in whom none of the criteria for a confirmed diagnosis were met AND who did not have a suspected infection | None | Infection of unknown origin |  |
| Unknown cause of fever | Patients with an unclear cause of fever: possibly infectious possibly a chronic cause or an underlying malignancy | None | Unknown |  |
| Non-infectious | Patients with a confirmed other cause of fever such as trauma. | Clinically of radiologically confirmed. | Non-infectious |  |
