# Supplementary material for: Infection Manager System (IMS) as a new hemocytometry-based bacteremia detection tool: A diagnostic accuracy study in a malaria-endemic area of Burkina Faso
Source: PLoS Negl Trop Dis. 2021 Mar 1;15(3):e0009187. doi: 10.1371/journal.pntd.0009187 (PMC7951874; doi:10.1371/journal.pntd.0009187)
Supplement: S2 Table — (DOCX) [file pntd.0009187.s004.docx]

**Supplementary Table 2. Overview of novel parameters determined by the XN haematology analyser series.**

| **Variable** | **Parameter** | **Unit** | **Description** | **Interpretation in clinical care** |
| --- | --- | --- | --- | --- |
| **Lymphocyte markers** | | | | |
| Total reactive lymphocytes | RE-LYMP (#)  RE-LYMP (%) | Cells/L  % | Activated B and T lymphocytes recognized by an increased fluorescence intensity compared to common lymphocytes | Metabolic activity in T-cells represents increased cytokine production. Early measure of cell-mediated immune response. |
| Antibody-synthesizing lymphocytes | AS-LYMP (#)  AS-LYMP (%) | Cells/L  % | Activated B lymphocytes (plasma cells) responsible for antibody synthesis. AS-LYMP is a proportion of RE-LYMP. | Antibody synthesizing B-cells in peripheral circulation represent a late innate or adaptive immune response. |
| **Neutrophil markers** | | | | |
| Immature granulocytes | IG (#)  IG (%) | Cells/L  % of WBC | Granulocyte precursors: consists of promyelocytes, myelocytes and metamyelocytes. | Granulocyte precursors are released into the periphery in case of shortage of granulocytes: in response to acute inflammation. Measure of innate immunity. |
| Neutrophil granularity index | NEUT-GI | SI | Complexity of neutrophil population representing cytoplasmic granulation and vacuolization. | Measure of toxic granulation and vacuoles increase in response to acute inflammation. Measure of innate immunity. |
| Neutrophil reactivity index | NEUT-RI | FI | Fluorescent intensity representing metabolic activity of the neutrophil population. | Measure of intracellular activity (e.g. cytokine production) increases in response to acute inflammation. Measure of innate immunity. |
| **Monocyte makers** | | | | |
| Activated monocytes | RE-MONO (#)  RE-MONO (%) | Cells/L  % | Proportion of monocytes with increased fluorescent intensity or complexity and monocytes with increased vacuolar activity | Increased fluorescent activity in monocytes represents metabolic activity, increased number of vacuoles are indicative of increasing phagocytic activity. Both increased in response to acute inflammation. |

* Adapted from Sysmex white papers hematology, October 2017
