# Supplementary material for: Infection Manager System (IMS) as a new hemocytometry-based bacteremia detection tool: A diagnostic accuracy study in a malaria-endemic area of Burkina Faso
Source: PLoS Negl Trop Dis. 2021 Mar 1;15(3):e0009187. doi: 10.1371/journal.pntd.0009187 (PMC7951874; doi:10.1371/journal.pntd.0009187)
Supplement: S3 Table — (DOCX) [file pntd.0009187.s005.docx]

**S3 Table. Results original IMS algorithm based on absolute numbers: Diagnostic accuracy for bacterial bloodstream infection including bacteraemia/malaria co-infections, bacterial infections and viral infections.**

| **Diagnostic accuracy for bacteraemia including bacteraemia/malaria co-infections.** | | | | | | |
| --- | --- | --- | --- | --- | --- | --- |
|  | **Less than 5 years**  **(n=333)** | | **5 years and older**  **(n=167)** | | **All ages combined**  **(n=500)** | |
|  | **IMS bacterial** | **IMS not bacterial** | **IMS bacterial** | **IMS not bacterial** | **IMS bacterial** | **IMS not bacterial** |
| **Confirmed bacteremia** | 33 | 17 | 59 | 8 | 90 | 25 |
| **Confirmed other infection** | 140 | 143 | 48 | 52 | 188 | 195 |
| Sensitivity | 66.0 |  | 88.1 |  | 78.6 |  |
| Specificity | 50.5 |  | 52.0 |  | 50.9 |  |
| PPV | 19.1 |  | 55.1 |  | 32.9 |  |
| NPV | 89.4 |  | 86.7 |  | 88.6 |  |
| ROC | 0.58 |  | 0.70 |  | 0.65 |  |
| **Diagnostic accuracy for all bacterial infections including bacteraemia and malaria co-infections** | | | | | | |
|  | **Less than 5 years**  **(n=345)** | | **5 years and older**  **(n=225)** | | **All ages combined**  **(n=570)** | |
|  | **IMS bacterial** | **IMS not bacterial** | **IMS bacterial** | **IMS not bacterial** | **IMS bacterial** | **IMS not bacterial** |
| **Confirmed bacterial** | 41 | 21 | 100 | 25 | 21 | 100 |
| **Confirmed other infection** | 140 | 143 | 48 | 52 | 143 | 48 |
| Sensitivity | 66.1 |  | 80.0 |  | 80.0 |  |
| Specificity | 50.5 |  | 52.0 |  | 52.0 |  |
| PPV | 22.7 |  | 67.6 |  | 67.6 |  |
| NPV | 87.2 |  | 67.5 |  | 67.5 |  |
| ROC | 0.58 |  | 0.66 |  | 0.66 |  |
| **Diagnostic accuracy for viral infections** | | | | | | |
|  | **Less than 5 years**  **(n=345)** | | **5 years and older**  **(n=225)** | | **All ages combined**  **(n=570)** | |
|  | **IMS**  **viral** | **IMS not viral** | **IMS**  **viral** | **IMS not viral** | **IMS**  **viral** | **IMS not viral** |
| **Confirmed viral infections** | 61 | 82 | 5 | 14 | 66 | 96 |
| **Confirmed other infection** | 14 | 188 | 8 | 198 | 22 | 386 |
| Sensitivity | 42.7 |  | 26.3 |  | 40.7 |  |
| Specificity | 93.1 |  | 96.1 |  | 94.6 |  |
| PPV | 81.3 |  | 38.5 |  | 75.0 |  |
| NPV | 69.6 |  | 93.4 |  | 80.1 |  |
| ROC | 0.68 |  | 0.61 |  | 0.68 |  |

PPV: Positive Predictive Value | NPV: Negative Predictive Value | ROC: receiver operating characteristic curve.
