# Supplementary material for: Infection Manager System (IMS) as a new hemocytometry-based bacteremia detection tool: A diagnostic accuracy study in a malaria-endemic area of Burkina Faso
Source: PLoS Negl Trop Dis. 2021 Mar 1;15(3):e0009187. doi: 10.1371/journal.pntd.0009187 (PMC7951874; doi:10.1371/journal.pntd.0009187)
Supplement: Attachment — Submitted filename: 201208_IMS letter of response.docx [file pntd.0009187.s007.docx]

Nijmegen, the Netherlands

December 5^th^ 2021,

To the editors of PLOS Neglected Tropical Diseases,

We would like to thank you for your thorough review of our manuscript and for giving us the opportunity to provide further details into the concerns and questions which were raised by the reviewers.

We have elaborated on all questions and remarks, please find below a point by point discussion of the different points raised. In case we did not agree with a recommendation made by one of the reviewers we explicitly mention this in the rebuttal.

We added line numbers and page numbers to facilitate the further review process. The line numbers provided in the rebuttal letter are based on the lines in the manuscript with “track-changes”. Based on the comment of reviewer #3 various textual changes were made to improve the English grammar and remove typographical errors. We furthermore adjusted the languages to American English instead of British English.

We hope that our responses will provide sufficient answer to the reviewers’ comments. Please do not hesitate to contact me for any remaining questions.

With kind regards, also on behalf of the research team,

Annelies Post,

**Reviewer #1**

*Query: Patients of ≥ three-month-old (age range can be used here).*

Reply: The age range (3 months to 90 years) has been added to the abstract (Page 2, line 46)

*Query: PCT performed significantly worse. This statement can be replaced by actual results.*

Reply: the actual result are now provided: The sensitivity, specificity and NPV of PCT at 0.5 ng/ml were lower at respectively 72.7% (95% CI: 60.4-83.0), 50.0% (95% CI: 37.4-62.6) and 64.7% (95% CI: 50.1-77.6) (Page 2, line 57-59)

*Query: Does this mean that IMS & CRP results were comparable?!*
Reply: In the various analyses IMS and CRP were either comparable, or IMS performed slightly better. Since there are multiple analyses done this is summarized in the sentence “the diagnostic accuracy of IMS was lower among malaria cases and patients < 5 years below five years old, but remained equal to- or higher than the accuracy of CRP.” (Page 2, Line 59-61)

*Query: “The diagnostic accuracy was lower among malaria cases ….” Be specific and provide complete sentences. diagnostic accuracy of IMS.*
Reply: The sentence was adjusted as follows: “The diagnostic accuracy of IMS was lower among malaria cases and patients < 5 years, but remained equal to- or higher than the accuracy of CRP.”

While we would like to include more data in the abstract this would inevitably cause the abstract to exceed the word limit. We therefore chose to show only the data which answer the primary aim of the study and report the rest of the data in the principal text. (Page 2, Line 59-61)

*Query: “Complete blood count is globally the most common requested laboratory examination.” Can be either removed or explained. Maybe it is related to statements mentioned in the last paragraph of discussion! to support the integration of hematology analyzer with the IMS algorithm.*
Reply: This sentence has been deleted since it does not serve a specific purpose here. The complete blood count test (CBC) provides a screening for a wide range of conditions and diseases and is the number one performed blood test in the US ref : <https://oig.hhs.gov/oei/reports/oei-09-15-00210.pdf>. A reference for this statement for other countries is more difficult to find but from experience we can say that CBC, together with malaria diagnostics, is also the most common test requested in Burkina Faso

*Query: “to reduce antimicrobial resistance formation” this can be rephrased.*

Reply: The sentence has been adjusted to: “Further studies are needed to see if the IMS can be safely used to guide initial antimicrobial treatment and help to reduce further spread of antimicrobial resistance.” (Page 3, Line 83-85)

*Query: Study design: Why the diagnostic accuracy of IMS among ≥ 5 years was considered the primary objective while in < 5 years was a secondary objective? The results involved both groups!! and the number of patients aged < 5 (331) was higher than those aged ≥ 5 years (135)!! Was this due to lower IMS accuracy reported among < 5 patients? but the design was decided before the commencement of data collection!!*

Reply: This design was based on previous experience with the IMS among adults whereas among children the IMS had not yet previously been tested. We used different age groups because for the adult population the development of the IMS was one step further advanced compared to the younger children. Since the normal distribution of blood cell lines and the immune response to pathogens among children differs from adults it was decided to treat these two as separate groups and analyze them separately. We also assessed the overall accuracy for all age groups combined. The explanation for the different age groups can be found in the supplementary materials.

(No changes to the text were made)

*Query: Study population and procedures: It is stated that “Patients were eligible if they ……… severe malnutrition with severe anemia.” Please revise this statement! In line with this statement, malnutrition prevalence presented in table 1 (41.6% in < 5 and 28.6% in ≥ 5 years) indicates severe malnutrition. And according to footnote, Z-score weight/height was used, i.e. the malnutrition here was the severe acute malnutrition (SAM). The reported proportions (41.6% & 28.6%) seem to be very high.!! Please revise.*

Reply: The text and Table have been clarified. The malnutrition mentioned in the Table represents a Z-score of -2 or lower; this therefore includes both patients with SAM and patients with MAM. A separate line for SAM has now been added to Table 1.

*Query: Study design: “among both patients below and over five years old as well as ….”. to include those aged 5 years, this can be changed to “**among both patients (i.e. below and ≥ 5 years old) as well as ….”*

Reply: this has been adjusted accordingly (Page 5, Line 129)

*Query: Study population and procedures: “malaria diagnostics”! These were mentioned in the supplementary file, but methods names can be mentioned between brackets.*
Reply: The used malaria diagnostics were added between brackets (Page 5, Line 148)

*Query: First paragraph and table 1: p-values are stated in the text but table 1 displays no values. A column can be added to Table 1 to present p-values.*

Reply: This comment was considered during the preparation of the manuscript. Since there are clear physiological differences between sick children below the age of 5 and over the age of five (both in vital signs and prevalence of infection) we decided that to provide significance levels for these variables would not contribute to the message. Since the only variables which can be compared between both groups were antibiotic/antimalaria pre-treatment and malnutrition percentages we opted to mention the resulting significance levels in the text instead of in the table.

No changes to the text were made.

*Query: In the text and tables 4-6, report all p-values to three decimals.*
Reply: In my experience the number of decimals provided depends on how exact the statistical analysis can be estimated and in many cases 3 decimals is simply not contributory to the message. I have now rounded down to 2 numbers after the first number over 0 (so 0.1 may have become 0.098, and 0.003 may have become 0.0032). I hope that this has improved the presentation of our results.

*Query: In the text, “…….. (n=55) led to a visually large (66.7% versus 75.0%) …but n= 58 in table 5!*
Reply: The text has been adjusted, thank you for pointing out this transcription error (Page 16, Line 322)

*Query: What was the patients’ age range (youngest & oldest)?*

Reply: The age range was 3 months to 90 years old. This has been added to the abstract and to the main text (Page 2, Line 46 and Page 8, Line 222)

*Query: Do you expect a difference between patients aged 5-15 years (n = 127) and those aged > 15 years (n = 338)?*

Reply: There will be a difference in prevalence of specific diseases. The rationale behind combining these age groups for the analyses is that the blood composition and immune response of children of 5 years and older is similar to that of adults. Therefore, the analyses could be combined leading to a larger n. We performed separate analyses for 5-15 and >15-year-old patients and results were comparable, though less reliable since they were all performed with a smaller n. In particular among patients of >15 years, the number of confirmed diagnoses was lower compared to the younger population.

*Query: The title stated “children” and “adult” but the results somehow did not clearly reflect such age grouping. The comparison was between < 5 and ≥ 5 years, with adults were included in the second group!!*

Reply: The title shall be adjusted. Please find the explanation for the comparison in the answer above.

*Query: Table 1A: “Median (IQR 25-75)” !! whats this?*

Reply: This means 25-75 interquartile range; it has been adjusted to “Median (Interquartile range (IQR))”

*Query: Text & table 2: “The sensitivity among ≥ five years old patients was higher than those < five years of age, while the latter patients had a higher specificity.” Why statistical tests were not used to compare the differences between both patients groups?*

Reply: Seeing as the development of the IMS was in a further advanced stage for the elder age group compared to the younger age group we decided not to compare them statistically. However, I have added some p-values in the text (Page 11, Lines 258-264)

*Query: Table 2: Bacteremia among malaria positive cases (n=268), with Less than five years (n=194) and Five years and older (n=74). How these 194 and 74 malaria positives can be extracted from table 1?*

Reply: Table 1 only presents the number of clinical malaria cases as proven by Malaria microscopy. Submicroscopic malaria parasitemia may or may not be the cause of malaria infection. The percentage of asymptomatic malaria parasitemia cases in the region is very high; up to 40% of the larger population. In order not to falsely classify patients as suffering from ‘clinical malaria’ patients with submicroscopic malaria were kept separate and only considered ‘clinical malaria’ in the following cases: 1) Malaria qPCR with > 50 p/uL and clinical picture of malaria or 2) a malaria qPCR of more than 0.05p/µL in a patient with the clinical picture of malaria that recently used antimalarials.

However, the presence of asymptomatic malaria parasitemia does influence the performance of the IMS, so all patients with a qPCR of >0.05 (with or without clinical malaria) were included as “with malaria parasitemia” in the accuracy analysis.

*Query: Add “n” of each of the 3 analysis groups, i.e. Bacterial infection among all cases; Bacterial infection among malaria negative cases and Bacterial infection among malaria positive cases. I have some confusion with Tables 4 and 6!!*

Reply: In Table 4 the numbers (n) per group are reported in the grey area immediately above the analyses. So respectively n=445, n=190, n=225. These values have also been added for Table 6.

*Query: First paragraph: “The relatively low specificity and PPV demonstrate that it is not (yet) suitable as a diagnostic for bacteremia.”*

Reply: While we do believe that the IMS can be used as a guide to exclude the presence of bacteremia (NPV) its specificity and PPV are currently not high enough to diagnose bacteremia; as a replacement for blood culture.

Query: Second paragraph: some limitations of the study findings are well-described in this paragraph but not mentioned in the limitation section!!

Reply: This is now acknowledged in the limitations section (Page 22, Line 429-430)

*Query: Third paragraph: “Both early malaria and bacteremia both …” remove the repeated “both”.*

Reply: This has been removed (Page 21, Line 390)

Query: Third paragraph: “….. some patients with asymptomatic malaria and undetected bacterial co-infection may have been falsely classified as malaria.”! Do you mean as malaria mono-infection? please rephrase to improve clarity.

Reply: This has been adjusted to ‘clinical malaria’ (Page 21, Line 384)

Query: Fourth paragraph: “… (a)symptomatic …” !!

Reply: This has been changed to “malaria parasitemia” (Page 21, Line 404)

**Reviewer #2**

*Query: Although citations have been given the described methods , it is important to briefly describe the steps involved, and provide information regarding the brand and catalogue number of materials and equipment used in the study (e.g. dyes, the brand of the device for CBC, CRP, PCT, microscopy etc). A table listing these materials/equipment with their sources & catalogue number is useful for readers that want to replicate the study.*

Reply: A full overview of the materials and equipment used, including brand names and methods of employ is provided as supplementary data 1. We previously included the information in the main text but this made the text significantly longer and more difficult to read. Besides, it was not explicitly needed to convey the message. Seeing as the supplements will be available online it was felt that providing this information as a supplement to those interested would suffice. Please let us know if you agree with this solution.

*Query: Page 14 “comparison with CRP and PCT”: The statement “Median CRP levels were the highest in bacterial infections……”. This is a little bit confusing after referring to Figure 3, as it seems like the higher levels were attributed to the Gram-positive bacteria and not the Gram-negative bacteria. Authors should discuss about this as well.*

Reply: The paragraph has been rewritten to further specify CRP and PCT results in different groups of infection. (Page 14, Lines 285-290)

*Query: Which bacteria is the median value provided in the text referring to? Or the value stated is based on analyses of all bacterial infections as a whole? If this were the case, the graph should be presented accordingly, or a separate plot is provided as a supplement.*

Reply: The first value referred to the median value of all bacterial infections combined. The text has been clarified and the figure has been adjusted according to your recommendations (see next query) (Page 14, Line 283-285)

*Concerning Figure 3: It is better to present the graph in scatter plots so that readers can see the distribution of the data points more clearly. I strongly recommend the authors to change the graph presentation to scatter plot. Also, in the text, the CRP value of viral infections was mentioned but it was not shown in Figure 3. This should be included, along with modifications on the figure legend.*

Reply: The graph has been changed into a scatterplot and a similar scatterplot for PCT has been added (Figure 3A-3D). A scatterplot for viral infections was not made, seeing as the objective of this paragraph was to show the overlap between malaria, bacterial infection and combined infection for CRP.

*Query: Other concerns/ issues that may affect the accuracy of this cellular immune response-based detection tool can be discussed more, such as application of this method on immuno-compromised groups (genetic and pathogen-induced), people with underlying conditions such as allergy, cardiovascular problems, diabetes mellitus, whose blood cellular components’ immuno-activity profiles may be different from those of normal, healthy individuals. These may be important points for medical workers from other countries to consider if they want to try this technique.*

Reply: In the current study we disregarded these factors seeing as our principle goal was to assess its capacity to differentiate bacterial infections from malaria and to assess if it could pick up on co-infections. The parameters used to create the IMS are however currently assessed for their capacity to differentiate infection from malignancy. Several studies have also been performed to assess the capacity of these parameters to detect bacterial infection among patients with known malignancies.

A segment on these further studies has been added to the discussion. (Page 22, Lines 413-418)

*Query: For individual harboring malaria parasites (and also all other individuals recruited actually), do the authors know if the subjects had taken any anti-malarial (or any other drugs) prior to blood collection? Some anti-malarials and drugs have immuno-modulatory properties, which may confound the results collected.*

Reply: We have the data on antibiotic and antimicrobial intake prior to inclusion; the percentages of patients using antimicrobial therapy are reported in Table 1. Unfortunately, we do not have reliable data on which antimicrobials exactly were used, therefore we cannot analyze the possible confounding effect of pre-treatment on the results. This concern has been added to the discussion as a limitation. (Page 22, Line 434 to Page 23, Line 436)

*Query: This method was previously used to differentiate between arboviral and bacterial febrile illness in Indonesia, detecting malaria in a well-controlled human experiment setting, as well as distinguishing between bacterial and viral infections in a pediatric setting. In this manuscript, the authors evaluated the efficiency of this method to differentiate between bacterial and non-bacterial acute febrile illness in a setting endemic to malaria, which could confound the method.*

Reply: This study was specifically designed to test if the IMS as previously designed in non-malaria endemic areas would also provide reliable results in a malaria endemic setting. With the current study we demonstrate that while the overall performance of the IMS is relatively good, even in case of concurrent malaria infection, the accuracy is indeed affected by the presence of malaria parasitemia; a conclusion which will be taken into account during the further development of the IMS technique.

*Query: "Age" seems to be an important factor to consider when using this method. What are the authors' opinions regarding application of this method in places with large elderly population?*

Reply: While age seems an important factor, the difference in accuracy was largely explained by the high prevalence of non-Typhoid Salmonella and the higher prevalence of malaria among young patients.

For populations

*Query: It would be good to discuss briefly possible ways to improve this detection tool.*

Reply: Please find a short additional paragraph added to the discussion (Page 22, Lines 413-418)

Query: Title page: The title does not quite highlight the core focus of this manuscript, which is the IMS. Here’s my suggestion for the authors' consideration: “Infection Manager System (IMS) as a new hemocytometry-based bacteremia detection tool in clinical settings: a diagnostic accuracy study in a malaria-endemic area of Burkina Faso”.

Reply: Thank you for the suggestion. We suggest to shorten the suggested title to: “Infection Manager System (IMS) as a new hemocytometry-based bacteremia detection tool: a diagnostic accuracy study in a malaria-endemic area of Burkina Faso”. We deleted” in a clinical setting” as this may be redundant. However, we have no problem to include it when the reviewer prefers to hold on to his/her originally suggested title.

*Query: Abstract; The clinical trial reference number in the abstract can be removed.*

Reply: The reference number has been deleted accordingly (Page 2, Line 47)

*Query: Author summary: please standardize the usage of abbreviation: “procalcitonin” should be “PCT” after the first mentioning of "procalcitonin"*

Reply: The word procalcitonin has been replaced with “PCT” throughout the manuscript, excepting the first times it was mentioned in abstract, authors summary, and main text.

*Query: Introduction, 2nd sentence: “AFI can be caused by a variety of pathogens- bacterial, viral, malaria-…”; “malaria parasites” or “Plasmodium spp.” may be more suitable to be addressed as “pathogens”.*

Reply: The sentence has been adjusted to: “Acute febrile illness (AFI) is an important health problem in sub-Saharan Africa (SSA). AFI can be caused by a variety of pathogens – bacteria, viruses, malaria parasites – which cause a non-specific clinical illness.“ (Page 4, Lines 87-89)

*Query: Introduction, last sentence of first paragraph: “the mortality of bacteremia is high and outcome depends on early recognition and treatment”, I suggest addition of “prognosis” before "outcome".*

Reply: The sentence has been adjusted accordingly (Page 4, Line 92)

*Query: Introduction, 2nd paragraph, 3rd sentence: “Even when severe malaria is diagnosed, antibiotics are commonly administered because of fear for co-occurring bactermia”. The word “co-occurring” used throughout the manuscript may be replaced with “concurring”. Can you provide a reference for this particular statement?*

Reply: co-occuring has been replaced for concurring throughout the manuscript. The sentence has been adjusted to become “Even when malaria is suspected, antibiotics are commonly administered because of fear for concurring bacteremia” and a reference has been added. (Page 4, Lines 97-99)

*Query: Introduction, 3rd paragraph, 4th sentence: the sentence should be started with a “The”.*

Reply: “The” has been added (Page 4, Line 109). In addition ‘the’ has been added before each use of ‘IMS’ throughout the manuscript.

Query: Please rephrase the third sentence of the methods section to make it clearer.

*Query: Table 4 (page 15), Table 5 (page 17) and Table 6 (page 19): Clear descriptions should be given regarding what the values within each bracket is representing.*

Reply: The descriptions (% (CI 95%)) have been added to the tables to clarify what each value represents.

*Query: Please standardize the usage of abbreviation throughout the text. “procalcitonin” should be “PCT”.*

Reply: This has been adjusted accordingly throughout the text.

*Query: Page 16, the 2nd sentence, typing mistake: “The sensitivity of CPR…..” should be “CRP” instead.*

Reply: Thank you for noticing, it has been adjusted (Page 16, Line 310)

*Query: The 6th sentence: the message may be clearer if it is split into two shorter sentences:* *In this sub-analysis, when compared with CRP, IMS had similar sensitivity but higher specificity. Both CRP and IMS showed higher sensitivity and specificity than PCT.*

Reply: The sentence has been adjusted according to the reviewer’s suggestion (Page 16, Line 316-318)

*Query: Page 20: 2nd sentence: “When using the ROC…..patients >5…”, “kindly add “years old” after the word “5”.*

Reply: The sentence was adjusted accordingly (Page 20, Line 336)

*Query: For section "IMS and CRP in a healthy control population" the subheading is misleading. The samples aren’t really “healthy controls” as some of them are infected with parasites, albeit without showing signs and symptoms. I suggest changing it to “symptom-free population”.*

Reply: The text has been adjusted accordingly (Page 20, Line 352)

*Query: In this segment as well, please give a brief concluding remark on the comparisons of different methods used.*

Reply: The following sentence has been added to the text “These results suggest that the IMS has a lower false-positivity rate in a symptom free population compared to CRP.” (Page 20, Lines 359-360)

*Query: Please use “cut-off” value throughout your manuscript when referring to “cut-off values”.*

Reply: This has been adjusted accordingly throughout the manuscript.

*Query: Page 21: The short paragraph, “Both early malaria…..in malaria patients”, followed by “iNTS is…..as well as by CRP”. A "bridge" is needed between these two sentences (first sentence referred to malaria, second sentence referred to INTS) to make the flow of the discussion content better.*

Reply:

*Query: Page 22, last sentence: “Both IMS and CRP…..”: the word “(a)symptomatic” can be removed. Also, in the same sentence, regarding the effect of parasitemia being stronger on CRP, is it backed by statistical analysis? If it is, please state it clearly in the text.*

Reply: We observe a large difference in specificity of CRP results between the patients with- and without malaria parasitemia; this difference larger in CRP compared to the IMS and results in a significantly higher specificity for the IMS compared to the CRP. On data inspection it was observed that the difference was indeed due to CRP levels generally being relatively high in malaria patients. We did not perform statistical analyses to compare CRP levels for the malaria versus the non-malaria group. We adjusted the sentence as follows “Both the IMS and CRP are influenced by presence of malaria parasitemia, though the large drop in specificity observed in CRP between patients with- and without malaria parasitemia suggests that the effect of parasitemia are stronger on CRP.” (Page 22, Lines 403-406)

*Query: In the same paragraph, this sentence may be better if the word “confounding” is added: “Most participants with positive CRP or IMS results were malaria microscopy positive, supporting the confounding effect of malaria on CRP and IMS results”.*

Reply: Thank you for this suggestion, it has been adjusted accordingly (Page 22, Line 409)

*Query: In supplementary data, page 4: a full description for RE-MONO/ RE-Mono should be given before using abbreviation, and the abbreviation used should be in consistent font format.*

Reply: The suggested alterations have been made to the text.

**Reviewer #3:**

Thank you for your review and your advice on improvement. Please find below a point to point reply to the various points raised. As some of the remarks were general to the whole text, I have added a list of the changes made based on each of your comments. I hope that these lists provide sufficient reply.

*Query: Throughout the whole manuscript, there are so many typographical and grammatical errors with confusing syntax. In addition, there are no page numbers and line numbers.*

Reply: Apologies for the missing page numbers and line numbers, these have been added. Please find below an overview of changes made based on the above-mentioned comment.
- The sensitivity, specificity and NPV of PCT at 0.5 ng/ml were lower at respectively 72.7% (95% CI: 60.4-83.0), 50.0% (95% CI: 37.4-62.6) and 64.7% (95% CI: 50.1-77.6) (Page 2, Line 57-59)

- Such a tool may guide clinicians in their decision the initiate of withhold antimicrobial therapy. (Page 3, Line 74-75)

- Standard microbiological techniques such as bloodculture were used as a reference to assess diagnostic accuracy (Page 3, Line 77-78)

- Further studies are needed to see if the IMS can be safely used to guide initial antimicrobial treatment and help to reduce further spread of antimicrobial resistance (Page 3, Line 83-85)

- The global increase in AMR has been decreed an imminent threat to global health by the World Health Organization (WHO). As a result, development of rapid diagnostics for differentiation between bacterial- and non-bacterial AFI has become a priority (Page 4, Line 101-103)

- Here we describe the diagnostic accuracy of the IMS to detect bacteremia and other bacterial infections in patients with AFI in a malaria endemic area in Burkina Faso (Page 4, Line 114-116)

- This leads to further differentiation of cell lineages (see also **Supplementary Table 2)** (Page 6, Lines 171-173)

- The IMS reports ‘Inflammation of unknown origin’ when inflammation is flagged but none of the likelihood scores match a decisive etiology. (Page 6, Lines 177-179)

- The blood samples were analyzed to assess how frequently IMS flagging inflammation was found among a healthy population with and without malaria parasitemia. Results were compared with plasma CRP levels (Page 7, lines 193-195)

**- Table 3** specifies the cases in which the IMS flagging for bacteremia was false negative and in which IMS flagging for bacterial infection was false positive. (Page 13, Lines 269-270)

*Query: There are no description for figure legends.*

Reply: The figure titles are provided in the text. Please find them at the appropriate sites in the text after the figures are first mentioned. Where appropriate a legend has been added.

*Query: Many abbreviations are not defined without full names.*

Reply: Abbreviations used in common medical practice such as HIV, PCR, DNA and RNA were indeed not defined, since it is assumed that the audience is familiar with these terms. All other abbreviations were defined at first use in abstract, authors summary and main text.

Based on this comment we have added the following abbreviation

- Systemic Inflammatory Response Symptoms (SIRS) (Page 5, Line 144)
- Polymerase Chain-reaction (PCR) (Page 13, Lines 274-276)
- TB has been changed to “Tuberculosis” (Table 1)

Query: Rational for “Newly diagnosed HIV infection was considered infection of unknown origin” should be described/further elaborated. Why?

Reply: Patients with a newly diagnosed HIV infection may present with a range of comorbidities. The immune suppressive effect of HIV causes opportunistic conditions (infections, malignancies) which is often reason to look for medical care. In cases in which a clear superinfection was found (e.g. bacteremia) the patient was included in the group of the superinfection. However, the rest of the newly diagnosed HIV patients was excluded since it was practically impossible to reliably place them into one of the categories.

*Query: The first line of Table 1; Median age (months/years) is very confusing. Less than 5 years ;18.8. Is this month? 5 years and older; 30. Is this year?*

Reply: This has been adjusted in Table 1. “Median age in months (<5 years) or years (≥5 years)”

*Query: The discussion can also be used by the authors to provide their thoughts/interpretation on the possible improvement of XN-30 equipped with IMS algorithm.*

Reply: A segment has been added to discuss possible ways to improve the IMS (Page 22, Lines 413-418)

*Query: Figure 4 describes ROC curves of the IMS likelihood score for bacterial infection compared to CRP and PCT for all groups. Authors should show different ROCs with or without malaria.*

Reply: A new series of ROC curves was made based on malaria infection; please find attached the novel Figure 4.
