# Supplementary material for: Infection Manager System (IMS) as a new hemocytometry-based bacteremia detection tool: A diagnostic accuracy study in a malaria-endemic area of Burkina Faso
Source: PLoS Negl Trop Dis. 2021 Mar 1;15(3):e0009187. doi: 10.1371/journal.pntd.0009187 (PMC7951874; doi:10.1371/journal.pntd.0009187)
Supplement: Attachment — Submitted filename: 210118_IMS letter of response.docx [file pntd.0009187.s008.docx]

Nijmegen, the Netherlands

January 17^th^ 2021,

To the editors of PLOS Neglected Tropical Diseases,

We would like to thank you for your interest in our paper, named “*Infection Manager System (IMS) as a new hemocytometry-based bacteremia detection tool: a diagnostic accuracy study in a malaria-endemic area of Burkina Faso”*. Please find below a point-by-point discussion of the various points raised. In the case where our opinion deviated from that of one of the reviewers, an explicit notion was added in this rebuttal.

We hope that our responses will provide sufficient answer to the reviewers’ comments and we are happy to provide additional information or answer any remaining question.

With kind regards, also on behalf of the research team,

Annelies Post,

MD, MSc Global Health

**Reviewer #2: Minor Revision**

*Query: Rules of using brackets: Do not use the same style of brackets when doing double-bracketing. Just like mathematics, there is such rule in English too, whose sequence of use is usually like this: {([ ])}. Please modify those double brackets in the tables accordingly.*

Reply: This has been adjusted accordingly.

*Query: Regarding the matter of not showing dataset of viral infections in plots of Figure 3 but only mentioned in text: Honestly, I don't like the idea of mentioning but not showing. However, if this is insisted and agreed upon by the editor, it is fine, but I strongly suggest you remodel your sentences in the manuscript, as readers will definitely expect the information (including the viral infections) to be available in the figures in its current form, which presents the 3 sets of data on the same ground.*

*My suggestion: Line 271: “….Among the different infections, median CRP levels of bacterial infections (88 mg/L, IQR 40 – 129; n = ?) were higher than those obtained from malaria cases. Interestingly, samples of viral infections recorded even lower CRP values (9 mg/L, IQR 3-26; n =?).” And similar style editing for the PCT description. If the viral infection dataset cannot be shown on the plots, the information should not be presented in the same sentence as the bacterial and malaria infections with the same tone of importance.*

Reply: We have adjusted the text according to your suggestion and added the bacterial-malaria co-infections as a separate group. We have furthermore added a supplementary figure where the three groups (viral overall, bacterial overall, malaria overall and combined malaria-bacterial overall) are mentions for PCT and CRP.

*Query: In addition, the mentioned stats in the text should be accompanied by the n number as they seemed to be different from those datasets presented in the graphs, and there are 3 groups of bacterial infections in the graphs, which readers would not be able to know which group the mentioned stats in the text were based on.*

Reply: We have added the n and have adjusted the text (please refer to Lines 270-287)

*Query: For the plots, you have datasets good enough to run statistical test for a more meaningful comparison, which is your objective in the manuscript. In the plots, please include a dotted straight line (parallel with the X-axis) to show the cut-off values of CRP and PCT that you used in respective graphs, so that readers can appreciate how different the values are between the normal and diseased states.*

Reply: Please find attached the new figure with the incorporated lines.

*Query: Please run a comparison test to make the claim in the text (line 275-278) meaningful.*

Reply: We have added several significance values to the text based on Kruskall-Wallis analyses. We previously refrained from statistical comparison since what we describe in this paragraph is such a minor part of our message. However, the results have been added on request.

*Query: Also in your plots and tables, I noticed that you used different concentration units. For example, in the graph, CRP is presented in mg/dL but in the table, it is in mg/L. Likewise for PCT, µg/L is used in the plot but ng/ml is used in table (although in this case, both units mean the same amount). Please be consistent throughout the manuscript (in text, table and figures).*

Reply: The CRP unit ought to be mg/L throughout. PCT in the plot has been adjusted to ng/ml like in the text.

*Query: Authors had the tendency to start a paragraph/ sentence with the words “Figure/ Table”. These look like a repeat of descriptions for the figure/ table legends. Please rephrase them to improve the manuscript. For example, line 315 can be modified like this:
“….We also investigated the impact of malaria infection on the diagnostic accuracy of these methods (Figure 4).* *For patients without malaria (parasites not detected from peripheral blood via light microscopy), IMS and CRP had comparable accuracy, whereas in patients with malaria, IMS was more accurate than CRP. Of note, PCT was the least accurate in both settings…..”.*

Reply: The comment has been adjusted though we used slightly different wording.

*Query: Please do not use the term “malaria parasitemia” as used in line 316. If the authors were trying to describe this as “patients harboring parasites detectable via microscopy”, kindly consider the above suggestion of rephrasing. In addition, please change other parts with similar style of presentation accordingly (Line 244, 258, 295).*

Reply: Here, our opinion deviates from the opinion of the reviewer. “Malaria parasitemia” is a frequently used term in literature. According to Oxford dictionaries “*Parasitemia is the demonstrable presence of parasites in the blood*.” We refer to patients as “with parasitemia” in case they have parasites detected in their blood by qPCR (which is one of the most sensitive ways of detecting malaria parasites), irrespective whether the parasitemia is cause of disease or not. This was done consciously since it is well known that asymptomatic malaria may have immunomodulatory effects and may therefore influence the results of our diagnostic technique. We therefore feel that the term we use is appropriate and suggest not to change it.

*Query: There are some issues of spacing and usage of punctuation marks throughout the manuscript (e.g. line 40, 73, 81, 94 to name a few). Please correct them.*

Reply: The manuscript was re-read several times and the United States English language and spelling check was run again. The text was adjusted accordingly.

*Query: Sentence at line 103: “…..which IS used to create……”*
Reply: The sentence has been adjusted accordingly

*Query: Line 242: please remove a redundant set of “with and”*
Reply: The sentence has been adjusted accordingly

*Query: Line 406: please remove the redundant “simultaneously”*

Reply: The sentence has been adjusted accordingly

Query: Also at Line 406: “…..decision making at costs similar to that of CBC alone.”
Reply: The sentence has been adjusted accordingly

*Query*: *Line 413: What is “Alinea”? Is it a typo? Please edit the sentence.*

Reply: the word “Alinea” has been replaced by paragraph.
